# Supplementary material for: ProteinShader: illustrative rendering of macromolecules
Source: BMC Struct Biol. 2009 Mar 30;9:19. doi: 10.1186/1472-6807-9-19 (PMC2672931; doi:10.1186/1472-6807-9-19)
Supplement: Additional file 1 — ProteinShader program without source code. This compressed file contains the complete ProteinShader program including associated libraries, but no source code. A README.txt file gives an overview of the ProteinShader distribution, and the index.html file in the help subdirectory has directions on getting started with the program as well as a set of tutorials. [file 1472-6807-9-19-S1.zip › ProteinShader-beta-0_9_4-binary/help/api/org/proteinshader/structure/Water.html]

Water (ProteinShader API)


|  |  |  |
| --- | --- | --- |
| **Overview** \| **Package** \| **Class** \| **Use** \| **Tree** \| **Deprecated** \| **Index** \| **Help** | |  |
| **PREV CLASS**   NEXT CLASS | **FRAMES**    **NO FRAMES**     **All Classes** |  |
| SUMMARY: NESTED \| FIELD \| CONSTR \| METHOD | DETAIL: FIELD \| CONSTR \| METHOD |  |


---


## org.proteinshader.structure Class Water

```
java.lang.Object
  org.proteinshader.structure.Residue
      org.proteinshader.structure.Water
```

**All Implemented Interfaces:**: IDTest, Visitable

---

``` public class Water extends Residue ```

This concrete subclass of the abstract class Residue stores
information on a water molecule in a PDB structure entry.

---

**Constructor Summary**

`Water(String residueID,
String chainID,
String modelID,
String structureID)`
  
          Constructs a Water with the requested residueID.


| **Method Summary** | |
| --- | --- |
| `void` | `accept(Visitor visitor)`             Accepts a Visitor and does a callback. |
| `Atom` | `getO()`             Returns the oxygen Atom (or null if not found). |

**Methods inherited from class org.proteinshader.structure.Residue**

`addNewAtom, getAtom, getChainID, getModelID, getResidueID, getStructureID, iteratorAtoms, numberOfAtoms, processID, toString`

**Methods inherited from class java.lang.Object**

`clone, equals, finalize, getClass, hashCode, notify, notifyAll, wait, wait, wait`

**Constructor Detail**

### Water

```
public Water(String residueID,
             String chainID,
             String modelID,
             String structureID)
      throws InvalidIDException
```

:   Constructs a Water with the requested residueID. When read from
    a PDB formatted file, the residueID is normally in a form such as
    "HOH 50" where "HOH" is the residue name and the integer is the
    residue sequence number. An leading or trailing whitespace will
    be trimmed from the residueId before it is stored.

    **Parameters:**: `residueID` - the ID of the Water.: `chainID` - the ID of the Chain the Water is associated with.: `modelID` - the ID of the Model the Water is associated with.: `structureID` - the ID of the Structure the Water is associated with. **Throws:**: `InvalidIDException` - if residueID is null or does not have at least one non-whitespace character.


**Method Detail**

### accept

```
public void accept(Visitor visitor)
            throws VisitorException
```

:   Accepts a Visitor and does a callback.

    :   **Specified by:**: `accept` in interface `Visitable` **Specified by:**: `accept` in class `Residue`
    :   **Parameters:**: `visitor` - the Visitor to do a callback with. **Throws:**: `VisitorException` - if an error occurs while an object is being visited.

---


### getO

```
public Atom getO()
```

:   Returns the oxygen Atom (or null if not found).

    :   **Returns:**: The oxygen Atom.


---


|  |  |  |
| --- | --- | --- |
| **Overview** \| **Package** \| **Class** \| **Use** \| **Tree** \| **Deprecated** \| **Index** \| **Help** | |  |
| **PREV CLASS**   NEXT CLASS | **FRAMES**    **NO FRAMES**     **All Classes** |  |
| SUMMARY: NESTED \| FIELD \| CONSTR \| METHOD | DETAIL: FIELD \| CONSTR \| METHOD |  |


---

# *Copyright © 2007-2008*
